# Ecosystem impacts by the Ancestral Puebloans of Chaco Canyon, New Mexico, USA

**DOI:** 10.1371/journal.pone.0258369

**Published:** 2021-10-27

**Authors:** David L. Lentz, Venicia Slotten, Nicholas P. Dunning, John G. Jones, Vernon L. Scarborough, Jon-Paul McCool, Lewis A. Owen, Samantha G. Fladd, Kenneth B. Tankersley, Cory J. Perfetta, Christopher Carr, Brooke Crowley, Stephen Plog

**Affiliations:** 1 Department of Biological Sciences, University of Cincinnati, Cincinnati, Ohio, United States of America; 2 Department of Anthropology, University of California, Berkeley, Berkeley, California, United States of America; 3 Department of Geography and GIS, University of Cincinnati, Cincinnati, Ohio, United States of America; 4 Archaeological Consulting Service, Ltd., Tempe, Arizona, United States of America; 5 Department of Anthropology, University of Cincinnati, Cincinnati, Ohio, United States of America; 6 Department of Geography and Meteorology, Valparaiso University, Valparaiso, Indiana, United States of America; 7 Department of Marine, Earth, and Atmospheric Sciences, North Carolina State University, Raleigh, North Carolina, United States of America; 8 Department of Anthropology, University of Colorado, Boulder, Colorado, United States of America; 9 Department of Geology, University of Cincinnati, Cincinnati, Ohio, United States of America; 10 Department of Anthropology, University of Virginia, Charlottesville, Virginia, United States of America; University at Buffalo - The State University of New York, UNITED STATES

## Abstract

The Ancestral Puebloans occupied Chaco Canyon, in what is now the southwestern USA, for more than a millennium and harvested useful timber and fuel from the trees of distant forests as well as local woodlands, especially juniper and pinyon pine. These pinyon juniper woodland products were an essential part of the resource base from Late Archaic times (3000–100 BC) to the Bonito phase (AD 800–1140) during the great florescence of Chacoan culture. During this vast expanse of time, the availability of portions of the woodland declined. We posit, based on pollen and macrobotanical remains, that the Chaco Canyon woodlands were substantially impacted during Late Archaic to Basketmaker II times (100 BC–AD 500) when agriculture became a major means of food production and the manufacture of pottery was introduced into the canyon. By the time of the Bonito phase, the local woodlands, especially the juniper component, had been decimated by centuries of continuous extraction of a slow-growing resource. The destabilizing impact resulting from recurrent woodland harvesting likely contributed to the environmental unpredictability and difficulty in procuring essential resources suffered by the Ancestral Puebloans prior to their ultimate departure from Chaco Canyon.

## Introduction

**Fig 1:**
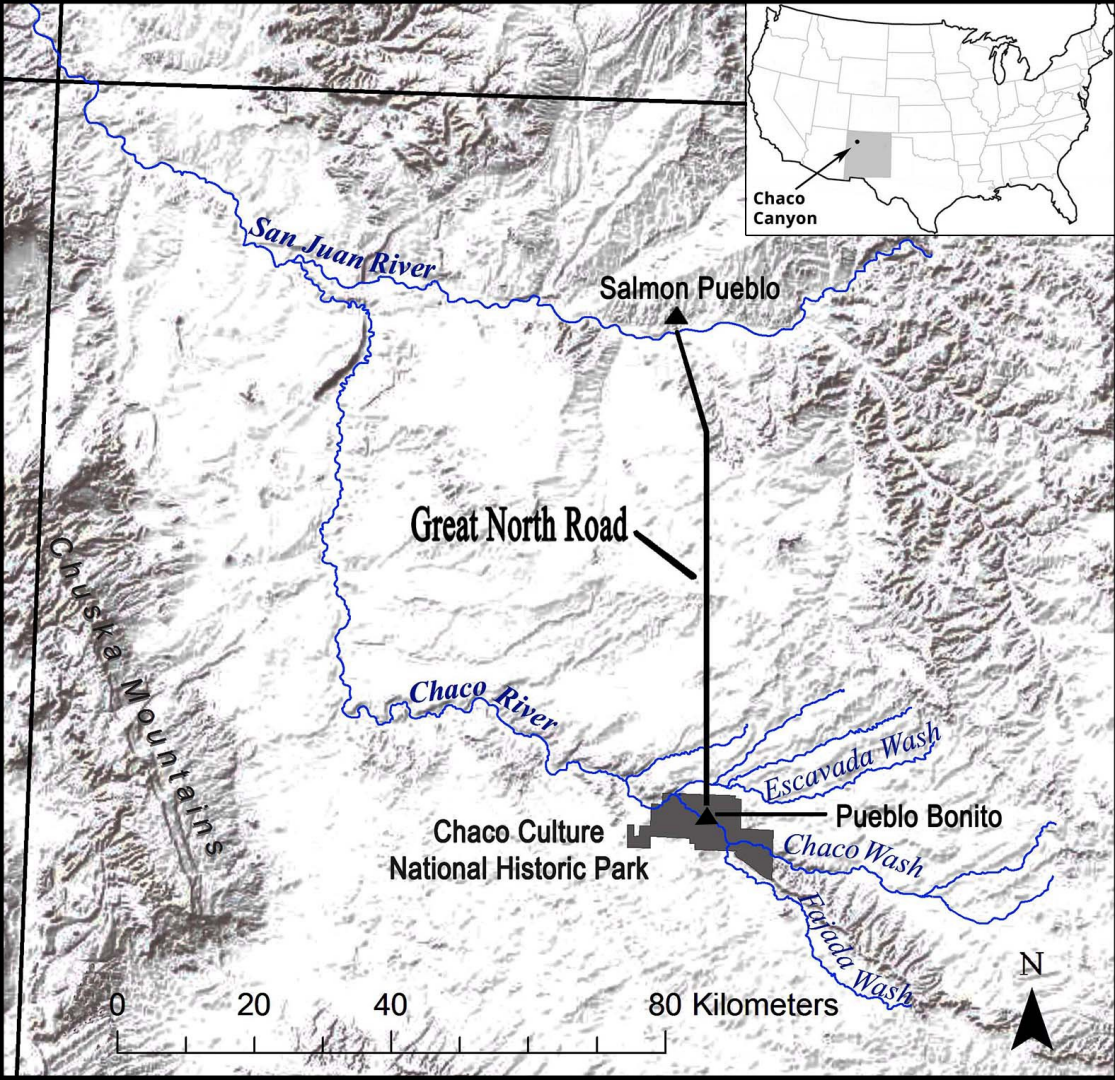
Map of the San Juan Basin in northwestern New Mexico. Map shows the location of Chaco Canyon in the Chaco Culture National Historical Park with related drainages and surrounding mountain ranges (Image created by Jon-Paul McCool and David Lentz. Reprinted from Fig 1 in [13] under a CC BY license).

The first human occupants of Chaco Canyon, located in what is now the southwestern region of the United States (Fig 1), arrived as early as Paleoindian times, ~10,000 BC (S1 Table in S1 File). During the subsequent occupation by Ancestral Puebloans beginning in Archaic times (3000–100 BC) and lasting until ~AD 1250 [1, 2], the canyon became a center of social complexity featuring a hierarchical society, intensive hydraulic agriculture, ceremonial activities, and the long-term maintenance of distant trade routes [2–5]. The ancient inhabitants of Chaco Canyon were best known for their iconic multi-story “great houses” constructed of stone masonry and huge timbers during the Bonito phase (AD 800–1130) [6], the period of great cultural florescence initiated by the Ancestral Puebloans, cf. [7, 8]. This period concluded with the emigration of many of Chaco’s residents from the canyon (S1 Table in S1 File), an outcome that has often been portrayed as a societal “collapse,” e.g., [9, 10], that coincided with prolonged droughts in the mid-12^th^ century [11]. Prior to the departure from Chaco Canyon, it has been suggested that unsustainable land-use practices resulted in woodland tree removal and bouts of erosion that had the effect of reducing the resilience of the landscape and likely exacerbated the ability of the Ancestral Puebloans to endure a period of extreme aridity [12].

Recently, scholars have challenged this characterization, stating there is no convincing evidence to support the scenario of deforestation caused by anthropogenic activities [14]. It has been the goal of our research to provide fresh insights into the sustainability of land-use practices in Chaco Canyon during the Ancestral Puebloan occupation. Here we add new data that reveal measurable changes in the pinyon juniper woodlands (PJW) that occurred before 600 BC when the food procurement system was transitioning from hunting and gathering to agricultural production. This shift in resource procurement enhanced the ability of the Ancestral Puebloans to sustain larger populations for several centuries, but by AD 1075–1100 it came at a cost of a major reduction of tree density in the local woodlands.

Pinyon and juniper trees provide many stabilizing influences on their woodland habitat. When trees, especially juniper which tends to be the most numerous of the two species, are removed, the result is soil erosion, altered hydraulic behavior, increased solar radiation on soil leading to greater evaporative losses [15] and loss of wildlife habitat [16]. Juniper trees have extremely deep (50 m or more) and extensive root systems that help to keep fragile soil layers intact [17]. If woodland tree cover is disturbed and erosion goes unchecked where the rate of soil loss outpaces the weathering of rock, the trajectory can become irreversible, resulting in severe habitat degradation [15].

**Fig 2:**
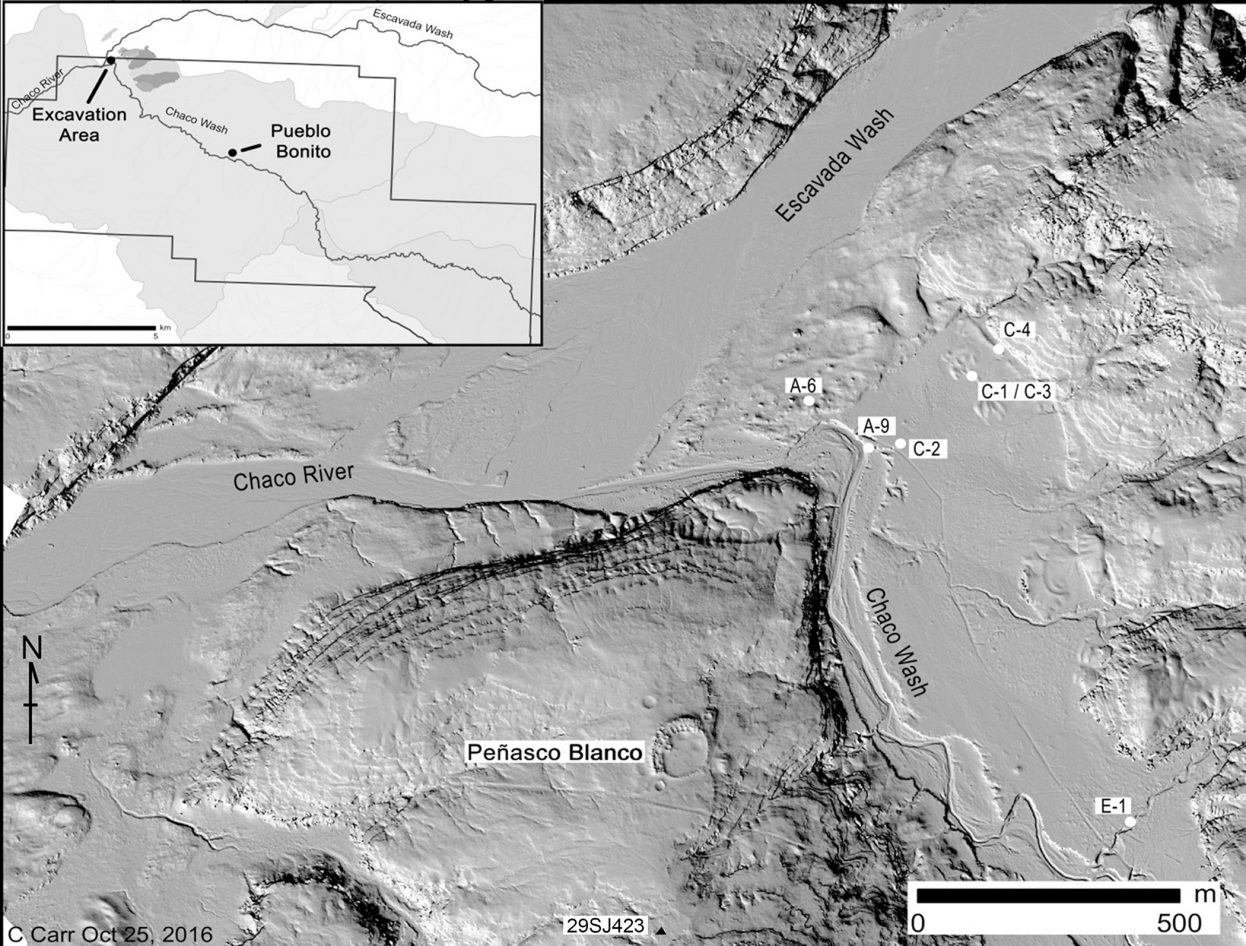
Hillshade view of the confluence of Chaco Wash and Escavada Wash. Map reveals the locations of excavation units discussed in the text (Image created by Christopher Carr and David Lentz. Base map [18]. Reprinted from [19] under a CC BY license, with permission from Vernon L. Scarborough, original copyright 2018).

Today the centerpiece of Chaco Canyon is a 32 km section of the Chaco Wash that flows intermittently from east to west. At its western end, Chaco Wash joins the Escavada Wash to form the Chaco River (Fig 2).

The north side of the canyon ranges from 1860 to 2010 m above sea level (asl) while the southern escarpment reaches heights of 2,165 m asl at the eastern end of Chacra Mesa [12], approximately 10 km southeast of the colossal Pueblo Bonito great house. Canyon soils display varying textures both spatially and with depth but have chemical and other edaphic qualities that are conducive to the growth of a range of crops [13, 19–21]. Mean annual rainfall within the canyon is 24.4 cm with average monthly temperatures varying between a low of -1.9°C in the winter to a maximum average of 22.6°C during the summer months [22].

The modern vegetation of the canyon is desert shrub-grassland with saltbush (*Atriplex canescens*) and greasewood (*Sarcobatus vermiculatus*) as the prevalent woody species on the alluvium-filled valley floor. In the uplands, or mesa top areas, sagebrush (e.g., *Artemesia tridentata*) and Mormon tea (*Ephedra* spp.) are the visually dominant plant species [23]. Occasional one-seed junipers (*Juniperus monosperma*) can be found on the canyon sidewalls, while a mixture of juniper trees and pinyon pine (*Pinus edulis*) occupies the north side of Chacra Mesa on the western edge of the canyon [23, 24]. Small riparian trees, such as willows (*Salix* spp.) and cottonwoods (*Populus* spp.) grow along the banks of the wash. At the higher elevations surrounding Chaco Canyon, particularly above 2,300 m asl in the adjacent mountain ranges reside significant stands of ponderosa pine (*Pinus ponderosa*) along with Douglas fir (*Pseudotsuga menziesii*), alpine fir (*Abies lasiocarpa*), Rocky Mountain white oak (*Quercus gambelii*), and blue spruce (*Picea pungens*) [24]. All of these tree species were extensively utilized by the Ancestral Puebloans of Chaco Canyon as they undertook the monumental task of constructing their great houses during the Bonito phase [25].

An understanding of the climate history of Chaco Canyon is an essential part of placing cultural change in an environmental context. Climate data going back to the Middle Archaic period reveal that from ~4000 to 2200 BC there was an interval of increasing aridity that favored the spread of the PJW into the canyon at the expense of the existing ponderosa pine forests [26]. The dryness continued until around 400 BC when a more stable, mesic period ensued (although drier and warmer than today’s climate) with both wet and dry intervals [24]. This period of low temporal variability, which coincided with the spread of agriculture in the canyon, lasted until around AD 750, when the intervals between minimum and maximum rainfall decreased [25]. Wet years were soon followed by notably dry intervals [25]. The period from AD 990 to 1130, which overlaps with the Bonito phase, was a time of increasing precipitation [11]. Following this period was a multi-decadal drought that lasted from AD 1130 to 1180 and coincided with the departure from the canyon by many Chacoan inhabitants. This drought was followed by a century-long mesic period that witnessed the re-occupation of Chaco Canyon by Mesa Verde-affiliated inhabitants [27], only to be followed by what has been called the “Great Drought” that lasted from AD 1276 to 1299 [11]. During this time, the remaining inhabitants emigrated from the canyon. The drought was followed by a wet spell in the 1300’s [11] and the climate from the 15^th^ century onward was similar to that of the 20^th^ century [24].

The purpose of this study was to investigate the relationship between the ancestral Puebloans and their surrounding vegetative and fluvial environments in a setting of climatic variability. During excavations we collected ancient plant specimens in the form of pollen and macrobotanical remains (seeds, wood, etc.) to help determine if and how the floral complexion of the landscape changed through time.

## Materials and methods

Our research began in 2013 and continued for three consecutive summer seasons with a focus on sediment coring, arroyo sidewall profiling, and excavations at both the southeast and northwest boundaries of the Chaco Culture National Historical Park. Our data came primarily from seven excavation units (or operations) that targeted irrigation canals, check dams, rincon drainages and agricultural terraces located in and around the Chaco Wash floodplain (Fig 2). Notably there were Basketmaker II and Basketmaker III (AD 500–700) sites on the nearby mesa-tops and in the canyon as well as a large great house, Peñasco Blanco (AD 900–1125), located on the mesa just south of our 2015 excavations [28, 29].

Thirty-seven macrobotanical samples plus twenty-one soil samples for pollen extraction were collected during the 2015 field season at Chaco Canyon. Charred macrobotanical remains were recovered by dry screening (0.25 inch mesh) and were analyzed in the Paleoethnobotanical Laboratory at the University of Cincinnati initially using light microscopy and subsequently using environmental scanning electron microscopy on a Philips XL30 ESEM housed at the Advanced Materials Characterization Center at the UC College of Engineering. Each charcoal sample was imaged from transverse and tangential sections, obtained by hand fracturing, to provide maximum morphological information about the wood to enable identification. Otherwise, paleoethnobotanical identification techniques were employed as described previously [30].

**Fig 3:**
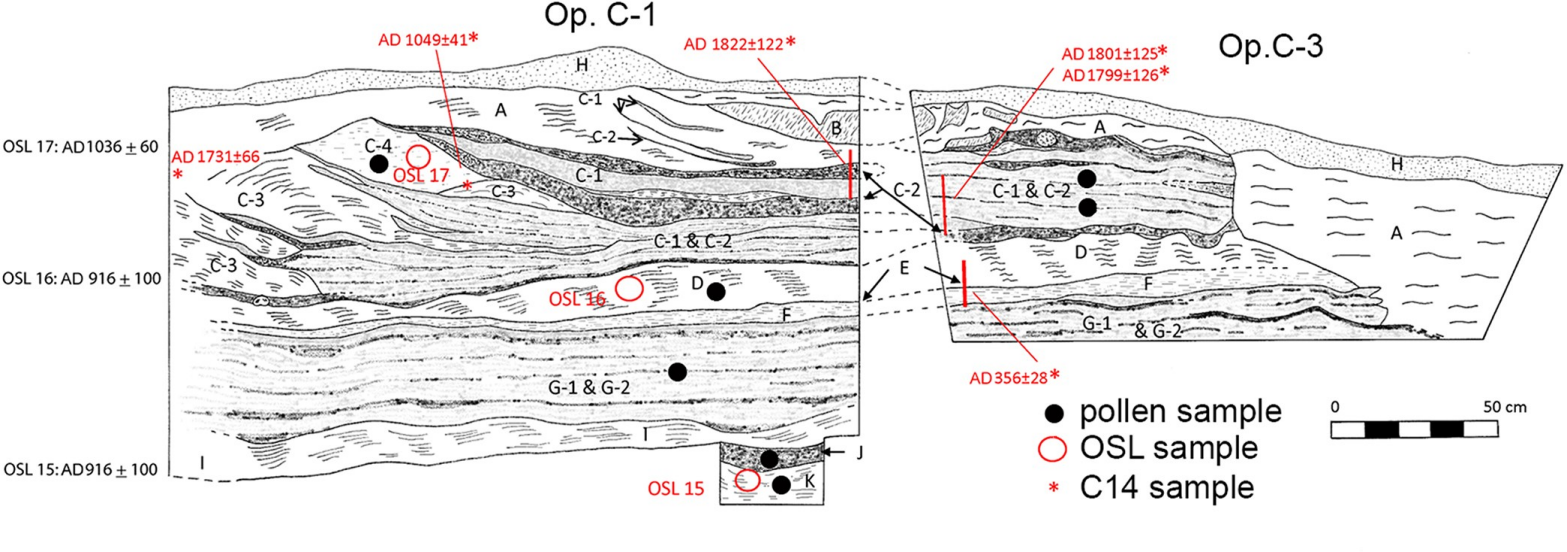
Stratigraphic profiles of Operations C-1 and C-3. (Image created by David Lentz. Reprinted from [19] under a CC BY license, with permission from Vernon L. Scarborough, original copyright 2018).

**Fig 4:**
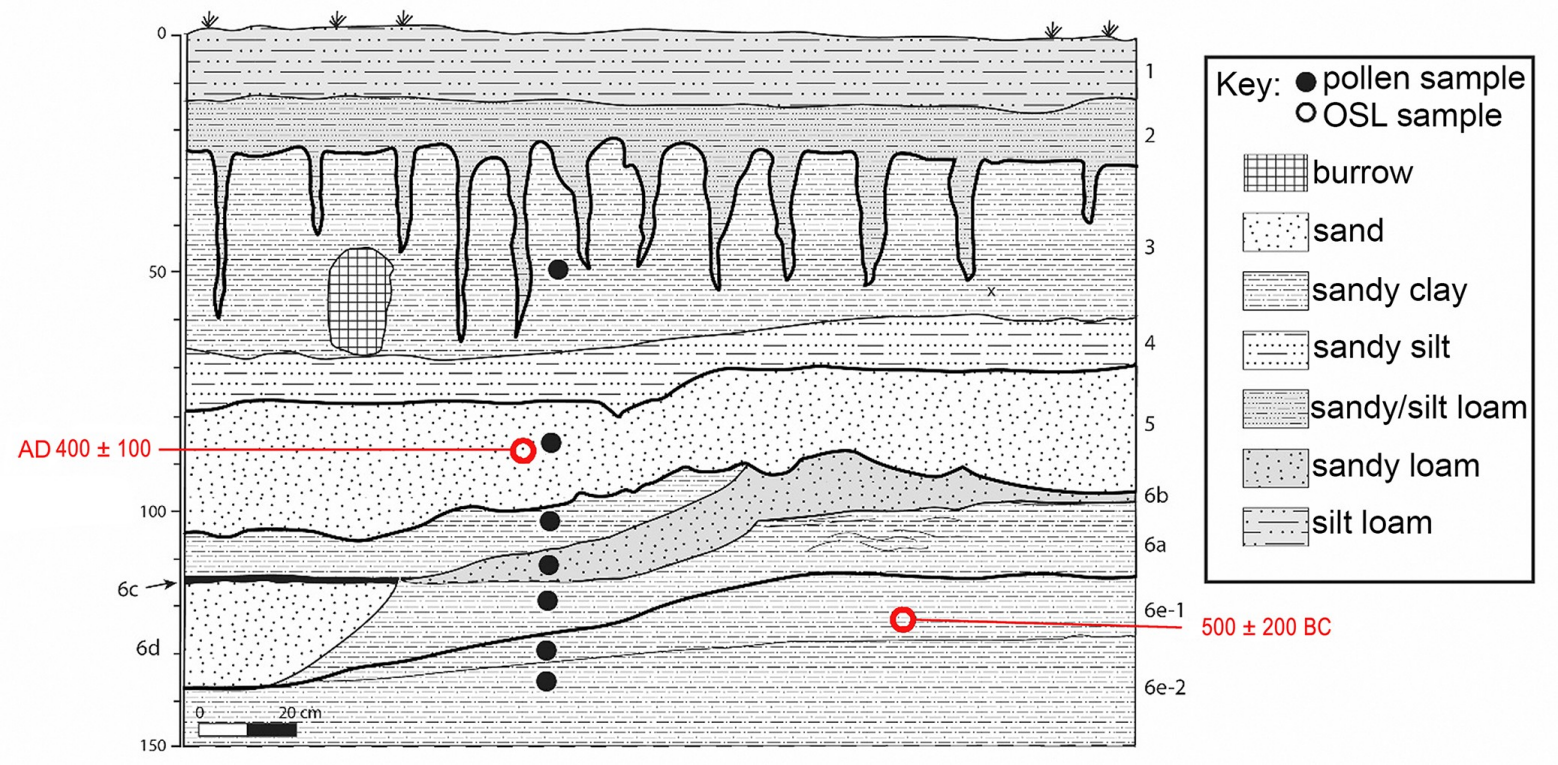
Stratigraphic profile of Operation C-2. (Image created by David Lentz. Reprinted from [19] under a CC BY license, with permission from Vernon L. Scarborough, original copyright 2018).

Soil samples designated for pollen analysis were collected in a stratigraphic column [31] from excavation profiles in Operations C-1/C-3 (Fig 3), C-2 (Fig 4), and C-4 and processed at the Texas A & M Pollen Laboratory using standard chemical extraction techniques [31]. *Lycopodium* spores were added to each sample to evaluate pollen concentration. Counts of 200 pollen grains were recorded for all samples with sufficient pollen following a methodology used in numerous previous studies (e.g., [30, 32, 33].

In addition to pollen content, soil samples were analyzed for isotopic signatures (δ13C), and chemical composition. Strata from excavations were chronometrically assessed using a combination of numerical dating techniques, viz., optically stimulated luminescence (OSL) (S2 Table in S1 File) and AMS radiocarbon (^14^C) analysis (S3 Table in S1 File). These data allowed us to evaluate alternative hypotheses relating to environmental impacts by Ancestral Puebloans at Chaco Canyon. Additional details of our research methods can be found in [33].

### Ethics statement

Because the excavations took place on federal property and the samples analyzed were derived from those sites, the project was conducted under permit number 15-CHCU-01 from the US National Park Service awarded to Vernon Scarborough. No additional permits were required for this study, which complied with all relevant regulations. The project was completed in cooperation with the Chaco Culture National Historical Park, the American Indian Advisory Council, the New Mexico Office of Archaeological Studies, and the Navajo Nation.

## Results and discussion

### Pollen record

**Fig 5:**
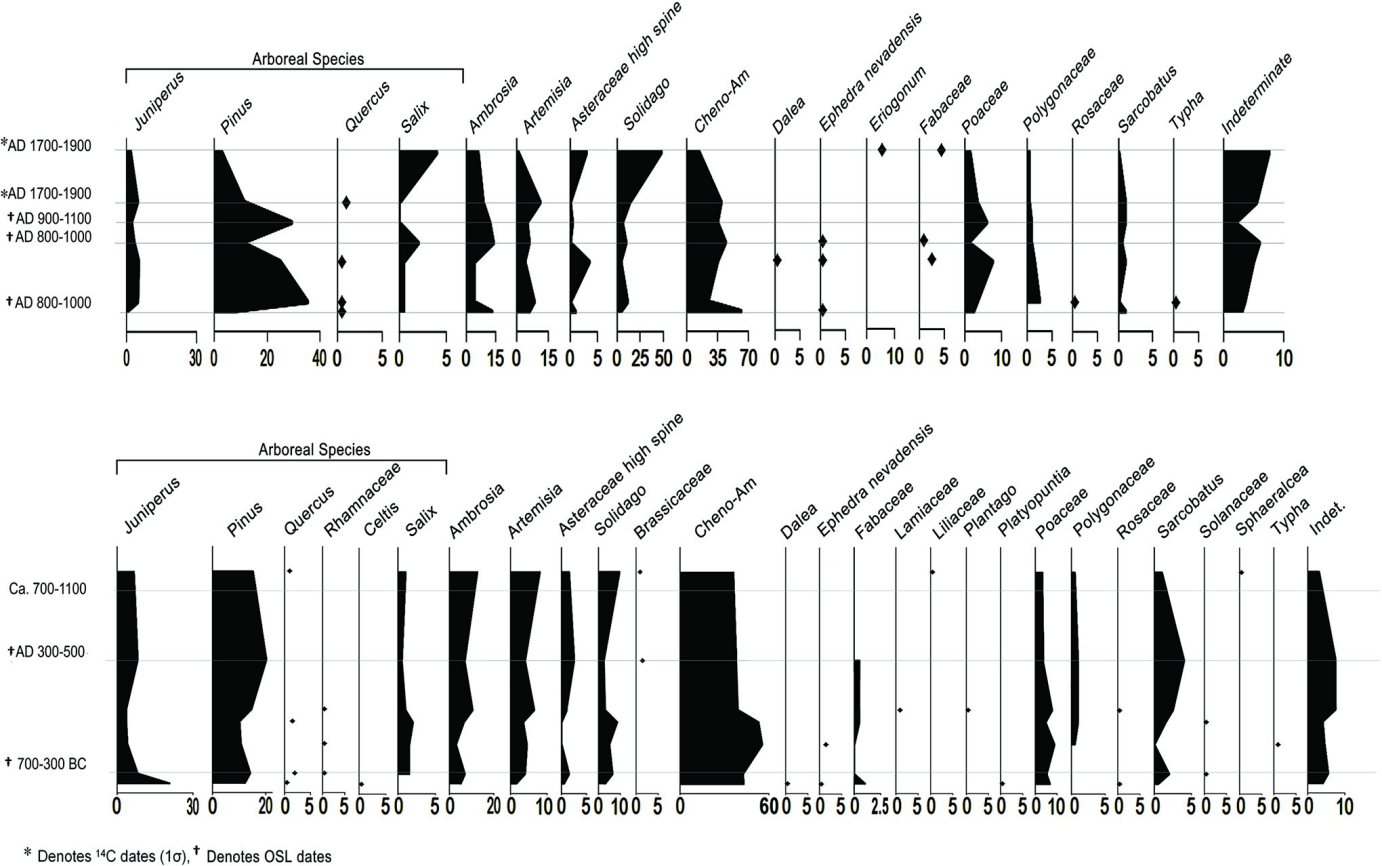
Pollen profiles. From Operations C-1/3 (top) and C-2 (bottom) (drawn by Cory Perfetta and David Lentz).

In brief, our pollen data indicate that there was a pinyon juniper woodland (PJW) in Chaco Canyon before 600 BC and reveal a sharp decline in juniper pollen thereafter. Before 600 BC the juniper and pine pollen readings (S4 Table in S1 File; Fig 5) were at 20% and 12% respectively, which is characteristic of a robust pinyon juniper woodland [34].

**Fig 6:**
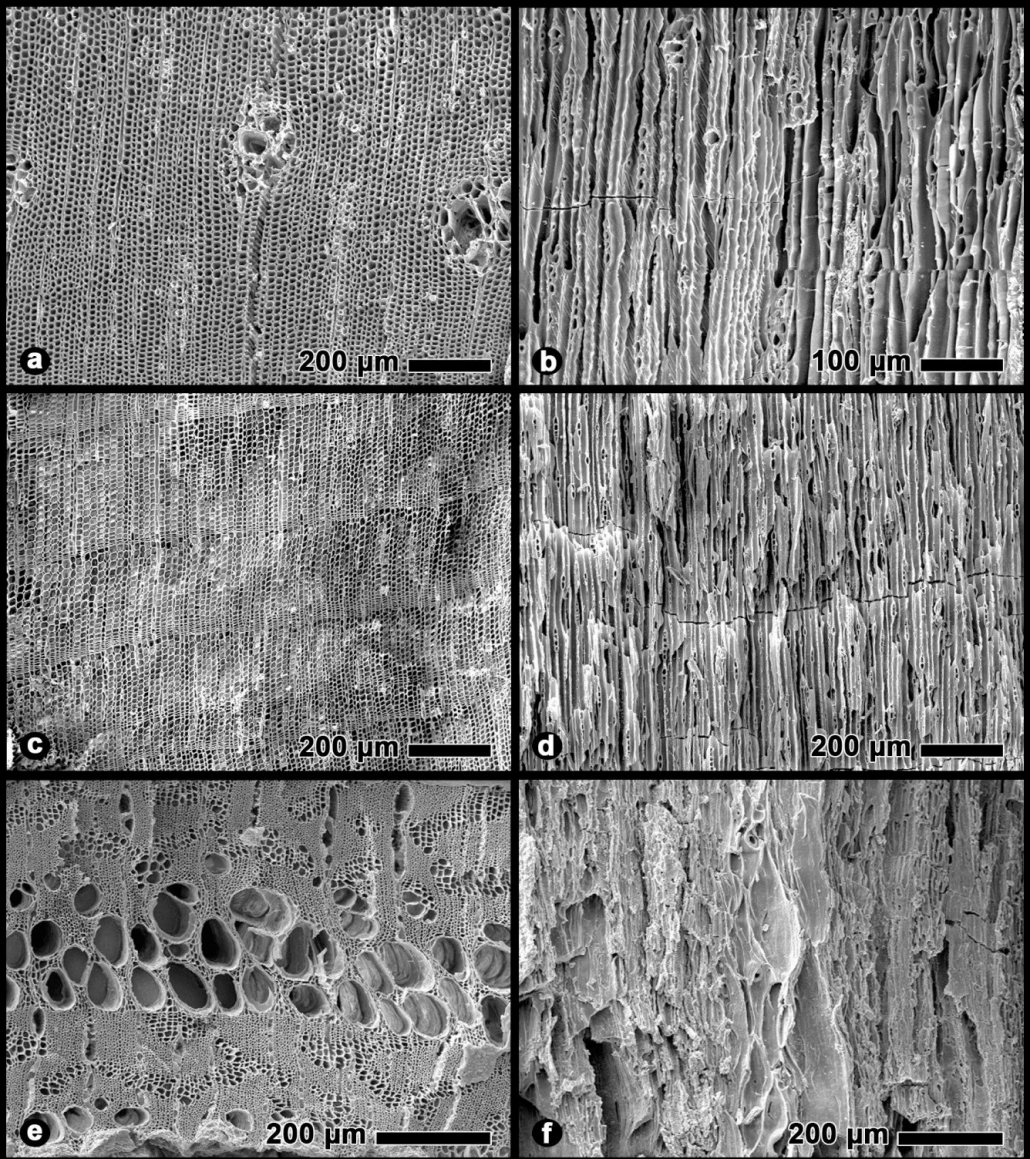
Scanning electron micrographs of carbonized wood specimens. Samples recovered from Chaco Canyon: a) transverse section of *Pinus edulis*, b) tangential section of *Pinus edulis*, c) transverse section of *Juniperus monosperma*, d) tangential section of *Juniperus monosperma*, e) transverse section of *Rhus* sp., f) tangential section of *Rhus* sp.

By AD 400, the juniper pollen percent dropped to 8% and then 4% in AD 600. By AD 1100 (S5 Table in S1 File; Fig 6), the juniper pollen dropped to 2%, which likely reflects infrequent juniper trees clinging to the sidewalls of the canyon or possibly ambient pollen blown in from surrounding montane areas. In effect, there were few, if any, juniper trees left in the canyon by the Late Bonito phase. These findings are in line with other studies that report juniper wood for construction was being imported into Chaco Canyon from the Zuni Mountains, 75 km away, as early as AD 850 [35], a sign that local juniper wood was in short supply by that time. From a broader perspective, our observed juniper population decline coincides with a period of erosion [24] in the canyon that began around 400 BC and continued until Pueblo II times (~AD 900–1000). Furthermore, our studies detected a period of high aggradation of sediments in the canyon from AD 900–1000 followed by other episodes of erosion in the 12^th^ century [19].

The pine pollen record is less clear in its overall trend as the pine pollen levels increase around AD 1000 then decline, possibly because pinyon was used differently than juniper and also because the pinyon pine pollen record is complicated by a potential influx of ponderosa pine pollen from the surrounding montane areas. Pollen, especially pine pollen with its air bladders that act like sails in the wind, can travel hundreds and even thousands of kilometers [36]. Accordingly, it is conceivable, even likely, that pine pollen could periodically waft downhill into the canyon from the montane pine groves, especially if storms occurred when the ponderosa trees were dispersing their pollen. Furthermore, our results reveal that the pollen record of greasewood, another fuel species favored by the Ancestral Puebloans [37], runs parallel to the juniper pollen trend. Greasewood pollen dropped down to 1% during the Bonito phase, and the pollen of willow, another useful woody species, completely disappeared during the same period. Because pollen records are created as percentages, if several anemophilous taxa are removed from the environment, it will tend to increase the percentages of species that are less impacted even if their populations are slightly reduced or stay the same. This helps to explain why the pine percentages go up as the pollen counts for juniper and other wind-borne pollen species go down. Other studies based on pollen from packrat middens in the canyon show a sharp decrease in pinyon pine after ~AD 800 [26]. A final note on our Chaco pollen results, as certain species decline in an area, other species are likely to take root and fill the void. That is exactly what we observe in the pollen counts as disturbance taxa, *Ambrosia* sp. (ragweed) and *Solidago* sp. (goldenrod), trend upward throughout this period.

The time horizon for the precipitous drop in juniper pollen (~500 BC) coincides with the Late Archaic occupation by Ancestral Puebloans [38] (S1 Table in S1 File), a time when maize (*Zea mays*) and squash (*Cucurbita pepo*) cultivation was becoming widespread [3, 39, 40], although the earliest pollen evidence for maize in Chaco Canyon dates to (~2414 BC) [41]. Common beans (*Phaseolus vulgaris*) were first found in the region at Tularosa Cave, a small settlement 300 km south of Chaco Canyon, with a calibrated AMS radiocarbon age of 270–770 BC [42, 43]. Similarly, beans are believed to have been in cultivation in Chaco Canyon around 500 BC [44]. Thus, it appears that the three major North American crop plants (maize, beans, and squash), formed the basis of incipient agriculture in the canyon by the end of the Archaic period. This was a significant development because these three plants, if grown and consumed together, offered a fully nutritious diet [45] and agricultural complementarity due to the nitrogen-fixing bacteria in the root nodules of beans that improved the success of all three plants [46]. Importantly, studies modeling the suitability of the soils and other factors relevant to the agricultural potential of Chaco Canyon revealed that the area had favorable conditions for the growth of this traditional suite of southwestern crops [13, 47, 48]. Note that the cultigens mentioned, especially beans, but to a lesser extent maize and squash, need to be boiled before they can be eaten. This required daily hearth fires which, in turn, created an inexorable demand for fuel that would have far outweighed the wood requirements for construction and pottery-making combined [30].

### Macrobotanical record

Macrobotanical remains from our Chaco Canyon excavations were recovered in the form of charcoal, or burned wood, and carbonized seeds. Of these two categories, charcoal was by far the most abundant in terms of weight and ubiquity (Table 1). Charcoal remains included one-seed juniper, sumac (*Rhus* sp.), pinyon pine (Fig 6), cottonwood, willow, and elderberry (*Sambucus* sp.) (S1 Fig in S1 File).

**Table 1:** Chaco Canyon macrobotanical remains from 2013–2015 excavations.

Family	Taxonomic Name	Common name	Plant Part	Weight (g)	Ubiquity	Op.	Field Sample	Depth (cmbd)	Chronology
**Seeds**									
Amaranthaceae	*Amaranthus* sp.	Amaranth	Sseed(1)	< .01	2.63%	A-6	FS100	108	~.AD 1930
Amaranthaceae	*Atriplex* sp.	Saltbush	Sseeds(2)	< .01	5.26%	A-6	FS100	108	~AD 1930
						C-2	Float	115	AD 306–506*
Amaranthaceae	*Chenopodium* sp.	Goosefoot	seed(1)	< .01	2.63%	A-6	FS100	108	~AD 1930
Portulacaceae	*Portulaca oleracea*	Purslane	seeds(2)	< .01	2.63%	A-6	FS100	108	~ AD 1930
Verbenaceae	*Verbena* sp.	Verbena	seed(1)	< .01	2.63%	C-2	Float	115	AD 306–506*
**Charcoal**									
various		Conifer	charcoal	0.49	7.89%	C-2	FS110	125	300–700 BC†
						C-4	FS115	107	
						C-4	FS174	153	3100–3700 BC†
Cupressaceae	*Juniperus* cf. *monosperma*	Juniper	charcoal	0.48	7.89%	C-4	FS117	28–37	~ AD 500–750
						C-4	FS116	98	100 BC-AD 50*
						C-3	FS98	60–92	AD 328–384*
Pinaceae	*Pinus edulis*	Pine	charcoal	1.96	10.53%	C-1	FS91	85	AD 800–1000†
						C-1	FS87	39–56	AD 900–1100†
						C-4	FS175	106	~AD 500–750
						C-2	FS113	100	AD 300–500†
various		Hardwood	charcoal	0.80	23.68%	A-9	FS102	185	
						A-9	FS103	240	
						A-9	FS105	115	
						C-1	FS90	53–57	AD 1018–1100*
						C-1	FS92	90	
						C-2	Float	115	
						C-3	FS99	91	
						C-4	FS173	138	AD 1026–1116*
						E-1	FS101	178	
Adoxaceae	*Sambucus* sp.	Elderberry	charcoal	6.75	18.42%	C-1	FS88	34–50	AD 1681–1925*
						C-1	FS93	55–71	AD 1665–1797*
						C-1	FS89	57–72	
						C-1	FS95	71–112	
						C-1	FS133	27–32	
						C-3	FS97	60	AD 1673–1925*
						C-3	FS96	42–55	AD 1676–1926*
Anacardiaceae	*Rhus* sp.	Sumac	charcoal	1.37	2.63%	C-2	FS108	115	AD 300–500†
Salicaceae	*Populus* sp.	Cottonwood	charcoal	1.49	2.63%	C-2	FS109	142	AD 708–776*
Salicaceae	*Salix* sp.	Willow	charcoal	0.35	2.63%	C-4	FS114	84	AD 500–700†
		Unknown	charcoal	3.09	2.63%	C-2	FS112	125	

Chronological assignments marked with an “*” were determined by AMS radiocarbon (^14^C) analysis. Chronological assignments marked with a “†” were determined by OSL dating. Samples marked with “~” are estimates based on stratigraphic contexts and adjacent chronometric assessments.

The seeds of saltbush, goosefoot (*Chenopodium* sp.), purslane (*Portulaca oleracea*) and verbena (*Verbena* sp.) were identified from various strata (S2 Fig in S1 File). Detailed descriptions of each taxon can be found in the Supporting information section. Pollen data were recorded from Units C2, C4 and C1/3 (S4 and S5 Tables in S1 File). The earliest readings begin around 600 BC and the most recent during the Ancient Chacoan period comes from strata dating to circa AD 1100.

The earliest charcoal from our study is coeval with the Basketmaker II phase when the transition from hunting and gathering to a greater dependence on maize-based agriculture was occurring [3]. This transition was followed by a marked population expansion within the canyon during the Basketmaker III period [49, 50]. These developments undoubtedly placed great pressure on local woodland resources. One of the largest Basketmaker III settlements (29SJ423) in Chaco Canyon which includes a large round ceremonial structure, or *kiva* [29, 51], is located just south of our excavations (Fig 2).

The charcoal specimens we recovered from canals and ancient agricultural fields likely were the by-product of the exploitation of these woody plants as fuel and construction material by the Ancestral Puebloan inhabitants of Chaco Canyon. Modern Puebloans distribute the ashes from hearth fires on their fields [52], and the ancient Maya did the same thing with the ashes from their hearth fires, probably as a means of soil amendment [53]. Extensive natural forest fires are infrequent in this area today, and probably the same was true in the past, because juniper and pinyon plants are widely spaced [54] and the fuel to support a spreading crown fire is generally not present [55]. Furthermore, there is evidence that the ancient cultures of the American Southwest undertook periodic low-intensity burning which may have protected the forests from crown fires [56] as well as improve forage for game [34]. Lightning strikes do occur, but these are generally limited to the struck tree [55]. Thus, the charcoal we recovered likely represents the remains from ancient fire pits.

Our macrobotanical data reinforce the pollen findings with the earliest juniper charcoal appearing at 100 BC and then disappearing after AD 750 (Table 1). The pinyon charcoal records follow a similar pattern, but begin and end slightly later. Pinyon charcoal first appears around AD 300 and ends at AD 1100 (Table 1). Carbonized wood remains of willow, cottonwood and sumac appear as early as AD 300 and are undetected after AD 750.

When considered together, the macrobotanical and microbotanical evidence described above provides valuable insights into the environmental changes that took place during the Ancestral Puebloan occupation of Chaco Canyon. The first observation to be drawn from our data set is that there was a sizeable pinyon juniper woodland in Chaco Canyon during Late Archaic times when early settlements by the Ancestral Puebloans were becoming established [40]. This assessment concurs with observations of other researchers who have largely relied on pollen and macroremains from packrat middens [12, 57]. Our pollen data are important because they independently document earlier findings about the nature of the vegetation in early Chaco Canyon and clearly indicate the waning of the juniper component of the PJW before ⁓500 BC, which is earlier than previously thought. Our macroremian data support the pollen data by showing that juniper was being burned at an early time and then declines steadily until the start of the Bonito phase. As part of this discussion, it is essential to clarify that the name “pinyon juniper woodland” is really a misnomer. The name suggests that pinyon is the dominant species in this woodland biome, yet juniper is the pervasively dominant species in the region today and was likely so in the past, as well, particularly in the area around Chaco Canyon [37].

The presence of pine and juniper among the Chaco Canyon charcoal remains, reinforced by our pollen data, support an earlier hypothesis that the Ancestral Puebloan occupants exploited the PJW through their extraction of timber for fuel and construction material [12, 57, 58]. However, we offer a more nuanced interpretation of extractive activities in the biome. Not only were juniper and pinyon unequally represented as woodland denizens with the former being more dominant and more numerous than the latter, but the pattern of exploitation at the hands of the Ancestral Puebloans impacted each of these two oligarchic species in different ways. Pinyon pine nuts were harvested and consumed as an important dietary component at least as early as the Archaic period [27]. Juniper cones, often called berries, also have had a long history of consumption, but more as a “starvation food,” i.e., something eaten only when nothing else was available [59]. Even though rising Chacoan populations began to rely more and more on domesticated foods during Basketmaker II and III times, they continued to exploit a wide range of wild gathered foods. Not until the mid-700s AD did domesticated foods provide the primary source of sustenance for the Chaco Canyon occupants [25]. Pinyon nutshells were common among the macrobotanical remains at Pueblo Bonito, Pueblo del Arroyo, Pueblo Alto, and Kin Kletso great houses, but juniper seeds were rare [59]. Thus, it seems clear that pinyon nuts were frequently used as a food source while juniper berries were not.

Both pinyon and juniper woods were used by the Ancestral Puebloans for fuel and construction material. At Pueblo Alto, one of the Chaco Canyon great houses that has been studied thoroughly for archaeological plant remains, juniper and pinyon together comprised 32% of the overall fuel use [59]. Of the two fuel species, however, juniper appears to have been the most often utilized. For example, at Pueblo Alto, 56% of the firepits and 73% of the warming pits contained juniper wood while 44% and 36% contained pinyon wood, respectively [37]. More dramatically, at the Spadefoot Toad site, a small Pueblo II settlement in the canyon occupied from AD 825–1025 [60], 40% of the firepits contained juniper while none contained pinyon [37]. At Salmon Pueblo (AD 1090–1280 [61]), a Chaco culture outlier (Fig 1) that also has been well-studied for plant remains, seven of eight hearths examined contained juniper charcoal while only one contained pinyon charcoal [62]. In terms of construction woods, local conifers (including both juniper and pinyon) made up 96% of the construction wood at Chaco Canyon sites during the Basketmaker III period, but thereafter high elevation woods were more commonly used [59]. At Pueblo Alto (AD 1020–1140), pinyon comprised 31% of the tree ring specimens used for floors and ceilings while juniper contributed 9%, suggesting that juniper was infrequently used for larger and medium-sized beams in the construction process during the Bonito phase [37]. In short, juniper was used chiefly for fuel and construction material while pinyon was used for food, construction and, to a lesser extent, fuel.

We have already made the point that fuel use in an agricultural society places the heaviest burden on wood reserves because fuel is needed daily for cooking otherwise inedible domesticated foods. Also, because pinyon nuts were a reliable food resource, the trees likely were protected to some extent, especially in the early years when wild foods were still an essential part of the Chacoan diet. The fact that juniper was used preferentially for fuel and pinyon likely was protected as a food source helps to explain why we see the dramatic decline in juniper pollen after 500 BC, but less pronounced changes in the pine portion of the pollen profile. It also explains why we see an almost complete loss of the juniper component of the PJW by AD 1000. In essence, the dominant species in this fragile biome became substantially reduced, if not eliminated, due to anthropogenic activities, and this disruption undoubtedly contributed to the bouts of erosion and concomitant sedimentation we observed in our stratigraphic analyses of canyon soil deposits [19].

### Human and climatic drivers of change

Wills and associates [14] argue that there was prehistoric deforestation of the local woodlands in Chaco Canyon and that it occurred earlier than had been previously thought. Our findings support this argument. However, Wills and associates also argue that there is no direct proof of human impacts that may have caused this deforestation. On this point, we do not agree. Data generated as part of the present study and other published studies, e.g., [12, 57], reveal converging lines of evidence that point to the same conclusion: there was a considerable reduction of the local woodlands resulting from the Ancestral Puebloan occupation of Chaco Canyon. The first set of evidence is the woodrat or packrat midden data demonstrating there was an extensive pinyon juniper woodland in Chaco Canyon in the Archaic period, probably as early as ~3000 BC [26]. Second, our pollen data also reveal an extensive PJW in the Archaic period followed by a sharp reduction in the principal species of the PJW in the Late Archaic period and thereafter. Juniper pollen counts and charcoal evidence continued to decline until sometime around AD 1100, indicating a drastic reduction of the PJW, and the cause was likely anthropogenic as climatic data indicate relatively mesic conditions during that time, especially during the Bonito phase [40, 63].

Moreover, the percentages of pinyon and juniper used in great house construction declined steadily from Pueblo I into the Pueblo III periods, being replaced by ponderosa pine, Douglas fir and spruce after AD 1000 [64–67]. Scholars with an architectural focus have argued that the Ancestral Puebloans shifted to the large highland trees, mostly from the nearby Chuska mountains [35, 65], because they needed their large and straight boles to serve as the primary beams (or *vigas*) to support the roofs of the great houses. Pinyon and junipers are less suitable for this kind of structural support because of their small girth and irregularly shaped trunks. The juniper and pinyon wood that was used for construction during the Bonito phase came from outside the canyon [35]. The Chacoans of Salmon Pueblo, however, used both juniper and pinyon for the construction of their great house [63], possibly because these woods were still available at this outlying community where less extraction pressure had been exerted on the local woodlands. In essence, the combined evidence suggests that the ancient inhabitants of Chaco Canyon utilized pinyon pine and juniper for construction and fuel when it was available. The decreased use of these woods at Chaco through time [25] was not because the trees were suddenly unappreciated for their qualities as fuel and timber, but because there were fewer of them readily available. These points provide strong evidence that there were significant human impacts on the PJW during the occupation of Chaco Canyon by the Ancestral Puebloans.

As an alternative to the anthropogenic explanation for the reduction of PJW during Chacoan times, Wills and his colleagues have suggested [14] that the juniper and pinyon pine woodlands of Chaco Canyon perished during one of the San Juan Basin droughts between AD 200 and 1300. PJW trees may be sensitive to changes in aridity and temperature, but overall, they are well-adapted to arid environments and are generally resistant to drought. A study of a multi-year drought in the Colorado Plateau found almost half of the pinyon trees (47%) still alive after four years of drought while 86% of the junipers survived [68]. The results of the Colorado Plateau study reinforce the idea that pinyon trees and especially junipers are drought tolerant. What we observe from our Chaco pollen data, though, is that the juniper populations remained intact through the Late Archaic period, a time of “great aridity” [24], then declined as the human population of Chaco increased during a time of more mesic conditions. If anthropogenic forces were not at play, then it would seem likely that the PJW would have expanded as a result of the more favorable climate of the first millennium, not contract as we observed. Also, it seems likely that if the PJW could survive the extensive aridification events of the Middle Archaic period, it likely could have survived the droughts that occurred between AD 200 and 1300.

Another response to the question about drought and its connection to the loss of PJW at Chaco Canyon is the fact that areas adjacent to Chaco Canyon have retained their PJW through time. Today, Chaco Canyon is surrounded by PJW (within 100 km) in areas of similar elevation [69, 70], but there is only sparse PJW in the canyon even though it was once abundant there. The areas around Chaco Canyon were thinly populated by Ancestral Puebloans [71] and the impact on the PJW appears to have been less substantial there. The best explanation for all these observations is that the Chaco Canyon woodland was depleted through centuries of exploitation and the canyon suffered extensive, anthropogenic degradation in conjunction with the Ancestral Puebloan occupation, with periods of drought having only a secondary effect. Similar results indicating significant reduction of local woodlands by Ancestral Puebloan agriculturalists in aggregated prehistoric settlements have been observed in the Mesa Verde area of southwestern Colorado, as well [72, 73].

Anthropological models of wood use derived from human behavioral ecology predict that human decision making in regard to fuel and timber gathering will be optimized [74]. In this situation, firewood of preferred quality and local proximity will be the first to be collected. Juniper is a preferred fuel among modern Puebloans [54, 75–79] and is sought after for that purpose. Furthermore, the principle of least effort, e.g., [80], predicts that the inhabitants of sedentary cultures, at least initially, will collect firewood from a limited radius near their habitation sites. For example, Navajo will only carry wood about 0.8 km to their dwellings [81]. This principle predicts that preferred fuel species will be selectively reduced as harvesting pressures increase, particularly in areas where removal is continuous over long periods [80].

If these models are applied to the Bonito phase Chacoans, then the gathering of firewood would have consumed more and more human energy as the occupants traveled further afield to meet basic fuel needs as their populations grew. This circumstance has an interesting parallel in resource depletion observed in recent studies of the procurement of wild game at Chaco during the Bonito phase. Based on isotopic and rainfall data it appeared that deer and other large mammals used as meat sources at Pueblo Bonito were obtained from distances greater than 40 km away [8, 82]. If local fuel and meat resources were both depleted and only available at great distances, this would have put tremendous pressure on the inhabitants to obtain essential resources.

Samuels and Betancourt [12] determined how much fuelwood a Puebloan family would need for all their cooking and heating needs. They calculated that 8.5 m^3^ of fuelwood would have been required to supply a family of five for one year. Widely accepted human population estimates for Chaco Canyon at the height of the Bonito phase (~ AD 800–1140) ranged from 2,100 to 2,700 inhabitants [2, 83]. If we take the lower estimate and multiply it by 340 years (the duration of the Bonito phase) we end up with a figure of over 1 million m^3^ of fuelwood needed by the Chaco inhabitants during the Bonito phase. It is hard to imagine that the already depleted, slow-growing, xeric-adapted PJW of Chaco Canyon could have survived this staggering level of usage. Thus, the scenario of a degraded PJW ecosystem where one of the oligarchic species was decimated during the Ancestral Puebloan occupation is supported by our archaeobotanical data and is strengthened by the observations of numerous episodes of erosion and aggradation seen in our archaeological excavations.

## Conclusions

Our findings agree with previous studies that hypothesize the Ancestral Puebloans had a significant impact on the PJW that once comprised the dominant vegetation of Chaco Canyon. Our results indicate that there was PJW in Chaco Canyon before 600 BC, and juniper, the dominant species of this vegetation type, was severely diminished by the time the Ancestral Puebloans departed the canyon. Because PJW is one of the most drought hardy biomes in North America and it endured through many episodes of severe drought for thousands of years prior to the advent of the Ancestral Puebloans in Chaco Canyon, juniper is unlikely to have died off because of droughts while juniper populations all around the canyon survived. Finally, our observations of increased sedimentation and aggradation in the canyon indicate that there was considerable erosion during the time of occupation by the Ancestral Puebloans [19]. These reports are in keeping with the prediction that the removal of a significant portion of the vegetation essential to holding the soil would lead to accelerated erosion. Our results support the concept of environmental change in Chaco Canyon during the Ancestral Puebloan occupation caused by a reduction of the local woodland biome. This circumstance forced the inhabitants to range widely for essential resources, especially fuel and game. These stressors, combined with drought conditions, likely contributed to the departure of the ancient occupants from the canyon.

Additionally, what we have observed from the Ancestral Puebloan existence at Chaco Canyon ostensibly was part of a larger global phenomenon of unprecedented acceleration of vegetation compositional change that began between 3 and 5 thousand years ago [84]. This trend of cultural shaping of landscapes was not unique to the Chaco Canyon settlers and was well underway in most terrestrial ecosystems by the time of the Bonito phase. Thus, the Ancestral Puebloans were participants in what has been described as an enduring legacy of terrestrial biosphere transformation engendered by human societies [85].

## Supporting information

S1 File (DOCX)
