# Supplementary material for: Ecosystem impacts by the Ancestral Puebloans of Chaco Canyon, New Mexico, USA
Source: PLoS One. 2021 Oct 27;16(10):e0258369. doi: 10.1371/journal.pone.0258369 (PMC8550600; doi:10.1371/journal.pone.0258369)
Supplement: S1 File — (DOCX) [file pone.0258369.s001.docx]

**[S1 File]**

**Ecosystem Impacts by the Ancestral Puebloans of Chaco Canyon, New Mexico, USA**

David L. Lentz^1*^, Venicia Slotten^2^, Nicholas P. Dunning^3^, John G. Jones^4^, Vernon L.

Scarborough^5^, Jon-Paul McCool^3,8^, Lewis A. Owen^6^, Samantha G. Fladd^7^, Kenneth B.

Tankersley^5,6^, Cory J. Perfetta^1^, Christopher Carr^3^, Brooke Crowley^5,9^, Stephen Plog^10^

^1^ Department of Biological Sciences, University of Cincinnati, Cincinnati, Ohio, United States of America

^2^ Department of Anthropology, University of California, Berkeley, Berkeley, California, United States of America

^3^ Department of Geography and GIS, University of Cincinnati, Cincinnati, Ohio, United States of America

^4^ Archaeological Consulting Service, Ltd., Tempe, Arizona, United States of America

^5^ Department of Anthropology, University of Cincinnati, Cincinnati, Ohio, United States of America

^6^ Department of [Marine, Earth, and Atmospheric Sciences](https://meas.sciences.ncsu.edu), North Carolina State University, Raleigh, North Carolina, United States of America

^7^ Department of Anthropology, University of Colorado, Boulder, Colorado, United States of America

^8^ Department of Geography and Meteorology, Valparaiso University, Valparaiso, Indiana, United States of America

^9^ Department of Geology, University of Cincinnati, Cincinnati, Ohio, United States of America

^10^ Department of Anthropology, University of Virginia, Charlottesville, Virginia, United States of America

⃰ Corresponding author

Email: [lentzdl@ucmail.uc.edu](mailto:lentzdl@ucmail.uc.edu)

**Methodology**

Following is a description of all of the excavation units, or operations, excavated by the project during three field seasons.

- - - - Operation A-6 was a test pit excavated in the intermittently active dune field often referred to as the “dune dam” in an area just north of the westernmost portion of Chaco Wash (e.g., Force et al. 2002).
- Operation A-9 was a carefully cleaned cut bank profile in the horseshoe bend area of the western portion of the Chaco Wash floodplain.
- Operations C-1 and C-3 (Fig 3) were combined to bisect much of an ancient canal elevated above the Chaco Wash floodplain. Optically stimulated luminescence (OSL) ages of AD 900 ± 100 were obtained from sandy alluvial strata D and K. These ages indicate that aggradation of sediments within the canal in its various phases appears to have occurred relatively rapidly. An OSL age of AD 1000 ± 100 (UC, OSL 2015-17) was obtained from a later sandy berm apparently constructed to narrow the canal and maintain its elevation. A radiocarbon age with a range of AD 1059 ± 41 (UCI 167243) was obtained from the same berm. Superadjacent canal sediments produced several radiocarbon ages ranging from AD 1665–1950. While it is possible that these ages represent more recent (possibly Navajo) reuse of the canal, these samples are from strata which experienced considerable bioturbation in the form of animal burrowing and root penetration – hence the carbon samples from these upper strata may well be intrusive (Scarborough et al. 2018).
- Operation C-2 (Figure 4) was a floodplain test excavation located 100 m northeast of Chaco Wash initiated as a geomorphic control for the canal excavation (Scarborough et al. 2018). The lower portions of the pit revealed a series of successive aggradation and scour episodes. The upper portion of the profile revealed a buried vertisol with deep cracks in-filled with later sediment.
- Operation C-4 cut into the berm and channel of a narrow canal that runs along the base of the rocky upland on the north side of Chaco Wash floodplain. This canal appears to have been fed by several rincons draining uplands immediately to the north, particularly the extensive bedrock outcrops along the northern escarpment of the canyon. The stratigraphy is complex and depths of samples are less relevant than stratigraphic position (Scarborough et al. 2018).
- Operation E-1 was a carefully cleaned cut bank profile in an entrenched portion of the Rincon 5 channel that cut through a section of the lower Chaco Wash floodplain.

***\Macrobotanical Remains***

All macrobotanical remains (Table 1) were retrieved by hand from soil matrices with the aid of a dry screen and examined under Wild Herbrugg M5 light microscope (6 to 50x). Plant remains were sorted into the categories of seeds and charcoal remains. Seeds were identified based on their size, shape, and texture and compared to reference collections and relevant texts (Delorit 1970; Martin and Barkley 1961; Musil 1978). Charcoal fragments were fractured into transverse and tangential sections to aid in the examination of their anatomical features (e.g., vessels, rays, and parenchyma) and mounted for imaging using a Philips FEI XL-30 Environmental Scanning Electron Microscope housed in the Advanced Material Characterization Center (AMCC) at the University of Cincinnati. Wood micrographs likewise were compared to paleoethnobotanical collections, reference books and an online database for identification (Brown 1928; Insidewood [https://insidewood.lib.ncsu.edu/search]). The distribution of each wood species was confirmed using several botanical references for the region (Gay et al. 1970; Little 1976; Martin and Hutchins 1980; Tidestrom and Kittell 1941; Wooton and Standley 1915).

Micrographs of the various seeds and charcoal fragments are presented in Figs 5, S1, and S2.

**S1 Fig. Scanning Electron Micrographs of carbonized wood specimens**. Recovered from Chaco Canyon: a) transverse section of *Sambucus* sp., b) tangential section of *Sambucus* sp., c) transverse section of *Populus* sp., d) tangential section of *Populus* sp., e) transverse section of *Salix* sp., f) tangential section of *Salix* sp.

**S2 Fig.** **Images of carbonized seeds**. From Chaco Canyon excavations: a) *Atriplex* sp. [10020 003]; b) *Verbena* sp. [10037-002]

Descriptions of coniferous plant families are presented first followed by dicotyledonous families.

CUPRESSACEAE

*Juniperus monosperma* (one-seed juniper)

Juniper is a common, scale-leaved evergreen that grows from 970–2300 m asl in elevation (Adams 2004) in much of northwestern New Mexico with growth habits that range from bushy shrubs to small trees (Preston 1961). Charcoal identified as one-seed juniper (Figure 5) was recovered from Operations C-3 and C-4. According to ethnographic accounts, juniper “berries” (actually fleshy cones) were traditionally consumed by Native American groups throughout the Southwest (Whiting 1966; Gallagher 1977; Harrington and Matsumura 1967). Archaeobotanical studies revealed that various ancestral Puebloans ate juniper berries (Doebley 1976; Lentz 1984, 2006) and the wood was highly valued for fuel (Adams 2006) because it burns cleanly with a high heat and has a fragrant odor (Peattie 1991). Owing to its strong resistance to

decay, it is also useful for construction (Peattie 1991). Juniper branches and posts were encountered in the recent re-excavations of canals around Pueblo Bonito and appear to have been used to construct a water management feature (Wills et al. 2016).

PINACEAE

*Pinus edulis* (pinyon pine)

Charcoal identified as pinyon pine (*P. edulis*) was recovered from Operations C-1, C-2, and C-4. In general, pinyon is a common tree in northwestern New Mexico and can be found at elevations between 1400 and 2600 m asl (Peattie 1991). The wood has been used for construction, firewood and the seeds (generally referred to as nuts) were roasted before consumption (Adams 2006; Whiting 1966). Both pinyon nuts and juniper berries have been frequently recovered at nearby ancestral Puebloan settlements (Adams 2006). Ethnographic studies reported that the Hopi obtained pine timber from over 60 km away for roof construction (Ebeling 1986). Pinyon pine was one of the most ubiquitous taxa within our study, encompassing nearly 11% of the samples.

ADOXACEAE

*Sambucus* sp. (elderberry)

Charred wood of *Sambucus* sp. (Figure 6) was found in relative abundance in operations C-1 and C-3. Today there are five species of elderberry in northwestern New Mexico (*S. neomexicana, S. neomexicana, S. pubens, S. microbotrys,* and *S. melanocarpa*) (Martin and Hutchins 1980). These small trees or shrubs grow in moist valleys and canyons, and along streams throughout northwestern New Mexico (Martin and Hutchins 1980; Wooton and Standley 1915). Elderberry plants are nitrogen-loving and, they thrive near locations with organic waste. Among extant Native American groups, elderberry flowers and fruits are widely consumed when cooked, but are poisonous if uncooked. The wood also was used to craft flutes such as the ones found in Room 33 of Pueblo Bonito (Pepper 1909; 1920).

AMARANTHACEAE

*Atriplex* sp. (saltbush)

Two carbonized saltbush seeds (Figure 7) were recovered from Operations A-6 and C-2 in our excavations. The former context suggests a modern association but the latter dates to AD 400 ± 100 (UC, OSL 2015-1). Saltbush is a shrub that grows several feet high on canyon floors, talus slopes and stream terraces. The seeds have been recovered in association with metates at Salmon Pueblo, a Chacoan outlier about 70 km north of Chaco Canyon (Figure 1), suggesting that saltbush seeds were a food resource (Adams 2006). Many Puebloan groups in the Southwest grind various species of saltbush into flour (Palmer 1878). Wild foods like saltbush increased the sustainability for the ancestral Puebloans because agriculture in this arid location was not always dependable (Plog and Hantman 1990). The Tewa and Hopi prefer the ashes of this wild plant when making dough for waferbread and corn tortillas to turn them blue (Robbins et al. 1916; Whiting 1966). Additionally, saltbush is used in construction and medicinal applications (Camazine and Bye 1980).

*Chenopodium* sp. (goosefoot)

The seeds of numerous species of *Chenopodium* spp. were consumed as components of the wild food diet of most Precolumbian Americans of the Southwest (Castetter 1935; Whiting 1966). Seeds of *C. watsonii* were common at the Pueblo Alto archaeological site in Chaco Canyon (Toll 1987). During our excavations, a single carbonized goosefoot seed was recovered from Operation A-6 in a modern Aeolian dune context.

ANACARDIACEAE

*Rhus* sp. (sumac)

Today, sumac trees and shrubs grow commonly in the dry hills and rocky slopes of New Mexico between 1200 and 2400 m asl. The sumac genus is represented by several species in the region (Martin and Hutchins 1980), including three (*R. microphylla, R. trilobata* and *R. glabra*) that produce edible fruits widely consumed by Native Americans in the Southwest (Ebeling 1986; Whiting 1966). A burned basket from the Chaco culture made of sumac was found in Tower Kiva at Salmon Pueblo (Webster 2006). Sumac charcoal was recovered from Operation C-2 in Chaco Canyon in a Basketmaker II context (AD 400 ± 100).

PORTULACEAE

*Portulaca oleracea* (purslane)

A single purslane seed was recovered in Operation A-6 in a modern Aeolian dune context during our 2015 excavations. Purslane seeds and leaves have been gathered by numerous historic groups in the Southwest, such as the Zuni, Hopi, Navajo, Acoma, and Laguna, for cooking and medicinal purposes (Adams 2006; Whiting 1966).

SALICACEAE

*Populus* sp. (cottonwood)

Cottonwood charcoal was identified in samples recovered from operation C-2 in a context that dated to AD 400 ± 100 (UC, OSL 2015-1). Cottonwoods are common trees along the canyons of northwest New Mexico and usually grow near water sources. Currently there are three species of cottonwood (*P. angustifolia, P. sargentii,* and *P. fremontii*) in northwestern New Mexico, and any of these could have been the wood source for the charcoal generated by the former ancestral Puebloan inhabitants. Puebloan peoples used cottonwood for fuel, construction, and in the manufacture of various artifacts such as drums, katsina figures and small boats. Historically, the wood from cottonwood has been used by Puebloans to make a variety of artifacts, e.g., kachina figures, drums and small boats (Dunmire and Tierney 1995; Whiting 1966). A carved artifact in the shape of a pinecone previously recovered from Chetro Ketl in Chaco Canyon was made from cottonwood (Vivian et al. 1978).

*Salix* sp. (willow)

Willow charcoal was recovered in Operation C-4 in a stratum that dated to AD 600 ± 100 (UC, OSL 2015-20). Various species of willow (*S. amygdaloides, S. exigua, S. irrorata, S. scouleriana* and *S. bebbiana*) have been observed along the stream banks and rivers in northwestern New Mexico (Wooten and Standley 1915). Owing to the broad arc of the annual ring seen in the micrograph of Figure 6, the specimen we examined was likely from a large tree such as *S. amygdaloides*. Willow wood was used by the Chacoan people to make baskets, arrow-shafts, paintbrushes, roof thatch, and applicator sticks (Vivian et al. 1978). Additionally, the Tewa use willow charcoal as a black body paint (Robbins et al 1916).

VERBENACEAE

*Verbena* sp. (verbena)

A single seed attributed to *Verbena* sp. was collected from archaeological excavations in Operation C-2 in a stratum that dates to AD 400 ± 100 (UC, OSL 2015-1). The genus *Verbena* is represented by dozens of species throughout New Mexico; these grow mainly on dry slopes, open plains, fields, and disturbed areas. Many Southwest groups incorporate these plants into their ceremonies and medicinal applications (Moerman 2002). *Verbena bracteata* and *V. imbricata* are the most common species in the Chaco Canyon area (Martin and Hutchins 1980).

***Palynological Evidence***

Results of the pollen analysis are illustrated in Figure 8. Juniper, pine (mostly *P. edulis*) and cheno-am (goosefoot and amaranth) pollen dominate the pollen rain prior to 500 BC. The juniper pollen percentage, at 20%, is especially noteworthy when compared to other studies of pinyon juniper woodlands (PJW) in the American southwest. For example, at Diamond Pond in the Great Basin, the juniper pollen rain was recorded at 14% at its peak, and this was interpreted as a robust juniper-dominated woodland (Miller and Wigand 1994). Accordingly, the percentages of juniper pollen in combination with the readings of pine pollen at Chaco (20%) can be taken as an indication of a healthy woodland in Chaco Canyon before 500 BC. After this time, however, juniper pollen declines sharply. Juniper pollen readings recover slightly around AD 400 then fall to 2% of the pollen rain around AD 1000 in the Bonito Phase. Junipers are anemophilous pollen producers and generate abundant wind-blown pollen (Little 1971) so this low pollen count indicates that there was little or no juniper growing in the canyon at that time. The juniper pollen observed may represent ambient pollen being blown in from the surrounding region or a few isolated juniper trees growing on the canyon walls.

Pine pollen fluctuates after 500 BC then declines sharply after AD 1000. The percentage of willow pollen (*Salix* spp.), an example from the riparian forest, also decreases around AD 1000. Conversely, cheno-am, grass (Poaceae) and Asteraceae (*Ambrosia*, *Artemesia* and *Solidago*) pollen all show increases through time after 500 BC, but subsequently drop in historic times, possibly due to the introduction of domesticated grazing animals. Interestingly, the pollen of *Sarcobatus* spp. (greasewood), representing another favorite fuel source of the ancestral Puebloans (Toll 1985), fluctuates between 500 BC and AD 400 then afterwards declines steadily into the Bonito phase, likely reflecting heavy fuel wood extraction.

**SI References Cited**

Adams, R.P. 2004. *Junipers of the world*. Bloomington, IN: Trafford. ISBN 1-4120-4250-X

Brown, H.P. 1928. Atlas of the commercial woods of The United States. Syracuse: The New York State College of Forestry.

Camazine, S. and R.A. Bye. 1980. A study of the medical ethnobotany of the Zuni Indians of New Mexico. *Journal of Ethnopharmacology* 2(4): 365-388.

Castetter, E.F. 1935. Ethnobiological studies in the American Southwest. The University of New Mexico Bulletin Whole No. 266: Biological Series Vol. 4(1).

Delorit, R. 1970*. An illustrated taxonomy manual of weeds seeds*. Wisconsin: Agronomy Publications.

Doebley, J.F. 1976. A preliminary study of wild plant remains recovered by flotation at Salmon Ruin, New Mexico. MA Thesis. Portales: Eastern New Mexico University.

Dunmire, W.W. and G. D. Tierney. 1995. *Wild plants of the Pueblo province: exploring ancient and enduring uses*. Santa Fe: Museum of New Mexico Press.

Ebeling, W. 1986. Handbook of Indian foods and fibers of arid America. Berkeley: University of California Press.

Force, E.R., R.G. Vivian, T.C. Windes and J.S. Dean. 2002. *Relation of ‘Bonito’ paleo-channels and base-level variations to Anasazi occupation, Chaco Canyon, New Mexico.* (Arizona State Museum Archaeological Series 194). Tucson: University of Arizona.

Gallagher, M.V. 1977. Contemporary ethnobotany among the Apache of the Clarkdale, Arizona area Cocina and Prescott National Forests. Albuquerque: Department of Agriculture.

Gay, C.W., D.D. Dwyer, and R. E. Steger. 1970. *New Mexico Range Plants*. Las Cruces, NM: Cooperative Extension Service of New Mexico State University.

Harrington, H. D. and Y. Matsumura. 1967. *Edible native plants of the Rocky Mountains*. Albuquerque: University of New Mexico Press.

InsideWood. Available at: http://insidewood.lib.ncsu.edu/search (accessed 25 Sept. 2020).

Lentz, D. L. 1984. Utah juniper (*Juniperus osteosperma*) cones and seeds from Salmon Ruin, New Mexico. *Journal of Ethnobiol*ogy 4: l9l-200.

- 2006. Juniper cones and seeds from Salmon Pueblo, in P.F. Reed (ed.) *Thirty-five years of archaeological research at Salmon Ruins, New Mexico: Vol 3, Archaeobotanical research and other analytical studies*: 853-858. Tucson: Center for Desert Archaeology.

Lekson, S.H. (editor).  2006.  The Archaeology of Chaco Canyon.  School of American Research Press, Santa Fe.

Little, E.L. 1971. Atlas of the United States trees volume 1. conifers and important hardwoods. Washington: United States Department of Agriculture.

- 1976. *Southwestern trees: a guide to the native species of New Mexico and Arizona*. Washington: United States Department of Agriculture.

Martin, A.C. and W.D. Barkley. 1961. *Seed identification manual*. Berkeley: University of California Press.

Martin, W.C. and C.R. Hutchins. 1980. *A flora of New Mexico*, Vol. 1-2. Germany: J. Cramer.

Miller, R.F. and P.E. Wigand. 1994. Holocene changes in semiarid pinyon-juniper woodlands. *Bioscience* 44: 465-474.

Moerman, D.E. 2002. *Native American ethnobotany*. Portland: Timber Press.

Musil, A.F. 1978. *Identification of crop and weed seeds*. Washington: United States Department of Agriculture.

Palmer, E. 1878. Plants used by Indians of the United States. *American Naturalist* 12: 593-606.

Peattie, D.C. 1991. *A natural history of western trees*. Boston: Houghton Mifflin.

Pepper, G.H. 1909. The exploration of a burial room in Pueblo Bonito, New Mexico, in *Putnam Anniversary Volume, Anthropological Essays presented to Fredrick Ward Putnam in Honor of his Seventieth Birthday: 196-252.* New York: G.E. Stechert and Company.

-1920. *Pueblo Bonito.* New York: Anthropological Papers of the American Museum of Natural History 27.

Plog, S. and J. L. Hantman. 1990. Chronology construction and the study of prehistoric culture change. *Journal of Field Archaeology* 17(4):448-51.

Preston, R. J., Jr. 1961. *North American trees*. Cambridge, MA: M.I.T. Press.

# Reed, P.F. 2004. The Puebloan Society of Chaco Canyon. Westport, CT: Greenwood Press.

Tidestrom, I. and Sister T. Kittell. 1941. *A Flora of Arizona and New Mexico*. Washington The Catholic University of America Press.

Toll, M. 1985. An overview of Chaco macrobotanical materials and analysis, in J. Mathien (ed.) *Environment and subsistence in Chaco Canyon, New Mexico*: 247-277. Albuquerque: National Park Service.

-1987. Plant utilization at Pueblo Alto: flotation and macrobotanical analysis, in J. Mathien and T.C. Windes (ed.) *Investigations at the Pueblo Alto Complex Volume III Part 2: 691-784*. Santa Fe: National Park Service.

Vivian, R.G., D.N. Dodgen and G.H. Hartmann. 1978. *Wooden ritual artifacts from Chaco Canyon, New Mexico: the Chetro Ketl collection.* Tucson: University of Arizona Press.

Webster, L.D. 2006. Worked fiber artifacts, in P.F. Reed (ed.) *Thirty-five years of archaeological research at Salmon Ruins, New Mexico: Vol 3, Archaeobotanical research and other analytical studies*: 893-1012. Tucson: Center for Desert Archaeology.

Whiting, A.F. 1966. *Ethnobotany of the Hopi.* Flagstaff: Museum of Northern Arizona.

Wills, W.H., D.W. Lova, S.J. Smith, K.R. Adams, M.R. Palacios-Fest, W.B. Dorshow, B. Murphy, J.O. Sturm, H. Mattson and P.L. Crown. 2016. Water management at Pueblo Bonito. *American*. *Antiquity* 81(3): 449-470.

Wooton, E.O. and P.C. Standley. 1915. *Flora of New Mexico*. Washington: United States Government Printing Office.

**S1 Fig. Scanning Electron Micrographs of carbonized wood specimens**. Recovered from Chaco Canyon: a) transverse section of *Sambucus* sp., b) tangential section of *Sambucus* sp., c) transverse section of *Populus* sp., d) tangential section of *Populus* sp., e) transverse section of *Salix* sp., f) tangential section of *Salix* sp.


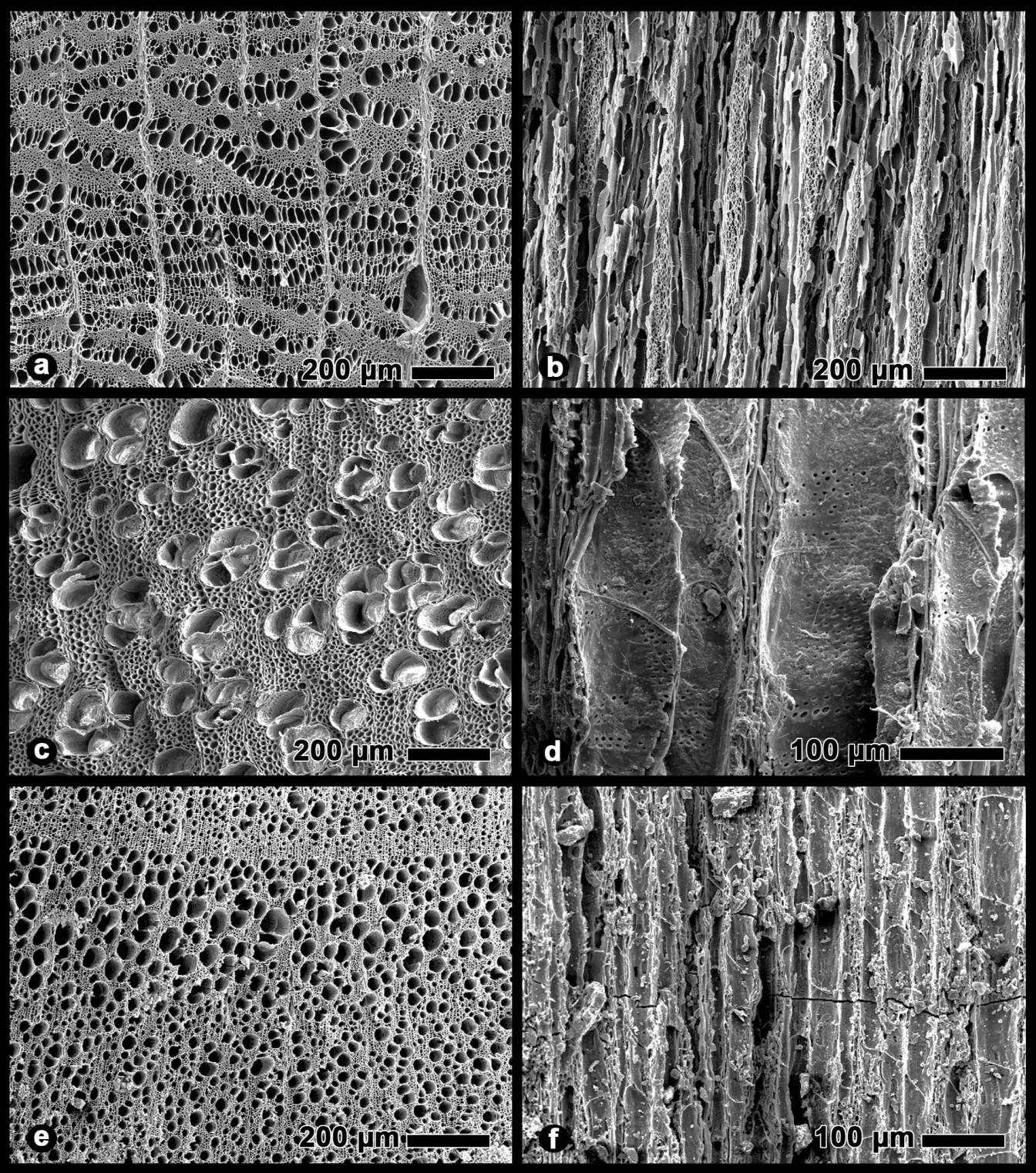


**S2 Fig.** **Images of carbonized seeds**. From Chaco Canyon excavations: a) *Atriplex* sp. [10020-003]; b) *Verbena* sp. [10037-002]


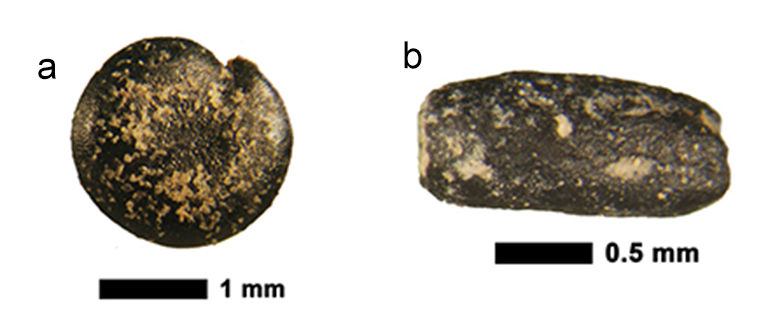


**Supporting information: Tables**

**S1 Table.** **Chaco Canyon chronology.** As described by Reed (2004) and Lekson (2006).

| **Pecos  Classification** |  | **Phase** | **Period** | **Cultural Activities** |
| --- | --- | --- | --- | --- |
| Late Pueblo III |  | Mesa Verde | AD 1200-1300 | Re-population by non-Chacoan occupants followed by final Precolumbian departure |
| Pueblo III |  | McElmo | AD 1140-1200 | Major Chacoan population decrease |
| Early Pueblo III |  | Late Bonito | AD 1090-1140 | Increase in Chaco Canyon population followed by a decrease, construction of great houses north of San Juan River |
| Late Pueblo II |  | Classic Bonito | AD 1040-1090 | Increase in population, first sign of kivas, large construction of great houses in the Canyon |
| Early Pueblo II |  | Early Bonito | AD 800-1040 | Row houses built on slabs above ground, increase in site frequency, early great houses appear |
| Pueblo I |  | White Mound | AD 700-800 | Large growth in number of storage cists, deeper pit houses with some aboveground room blocks |
| Basketmaker III |  | La Plata | AD 500-700 | Shallow pit houses, cists for storage |
| Basketmaker II |  | Brownware | 100 BC-AD 500 | More reliance on agriculture, more permanent storage and habitation, basketry, basin grinding slabs, ceramics |
| Late Archaic |  | - | 3000 - 100BC | Hearths, seasonal camps, horticulture with squash, maize and beans by around 500 BC, hunting and gathering important |

**S2 Table**. **OSL dating results**. Extracted from Chaco Canyon sediment as part of this project.

| **Sample**  **Number** | **Location**  **(°N/°W)** | **Operation** | **Depth**  **(cm)** | **OSL Age**  **(Ka)** | **Age in**  **Calendar**  **Years** |
| --- | --- | --- | --- | --- | --- |
| Chaco 2014-8 | 6.0/107.8 | OP. 100 | 100 | 0.9±0.04 | AD 1110±40 |
| Chaco 2014-9 | 6.0/107.8 | OP. 100 | 185 | 1.3±0.1 | AD 700±100 |
| Chaco 2014-10 | 6.0/107.8 | OP. 100 | 285 | 3.2±0.2 | 1200±200 BC |
| Chaco 2014-11 | 6.0/107.8 | OP. 100 | 320 | 2.9±0.1 | 900±100 BC |
| Chaco 2014-12 | 6.0/107.8 | A09 | 70 | 3.1±0.2 | 1100±200 BC |
| Chaco 2014-13 | 6.0/107.8 | A09 | 85 | 1.0±0.04 | AD 1000±40 |
| Chaco 2014-14 | 6.0/107.8 | A09 | 50 | 0.6±0.03 | AD 1400±30 |
| Chaco 2015-1 | 36.1/108.0 | C02 | 87 | 1.6±0.1 | AD 400±100 |
| Chaco 2015-2 | 36.1/108.0 | C02 | 122 | 2.5±0.2 | 500±200 BC |
| Chaco 2015-9 | 36.1/108.0 | A08 | 50 | 0.2±0.01 | AD 1860±10 |
| Chaco 2015-10 | 36.1/108.0 | A08 | 106 | 1.6±0.1 | AD 400±100 |
| Chaco 2015-15 | 36.1/108.0 | C01 | 117 | 1.1±0.1 | AD 900±100 |
| Chaco 2015-16 | 36.1/108.0 | C01 | 69 | 1.1±0.1 | AD 900±100 |
| Chaco 2015-17 | 36.1/108.0 | C01 | 28 | 1.0±0.06 | AD 1000±100 |
| Chaco 2015-20 | 36.1/108.0 | C04 | 68 | 1.4±0.1 | AD 600±100 |
| Chaco 2015-21 | 36.1/108.0 | C04 | 90 | 5.4±0.3 | 400±300 BC |

**S3 Table. AMS radiocarbon (^14^C) ages for samples discussed in the text.** The sample with an asterisk (*) was analyzed at the ICA laboratory in Sunrise, Florida. All others were analyzed at the Radiocarbon Laboratory at the University of California – Irvine.

| **Laboratory Sample Number** | **Field Sample** | **Composition** | **^14^C Age BP** | **Cultural Stage** | **Calibrated**  **Age (1σ)** |
| --- | --- | --- | --- | --- | --- |
| 167241 | FS-88 | *Sambucus* sp.  Charcoal | 170±20 | Historic Navajo | AD 1803±122 |
| 167242 | FS-88 | *Sambucus* sp.  Charcoal | 170±20 | Historic Navajo | AD 1803±122 |
| 167245 | FS-96 | Hardwood  Charcoal | 180±20 | Historic Navajo | AD 1801±125 |
| 167246 | FS-97 | *Sambucus* sp.  Charcoal | 185±20 | Historic Navajo | AD 1799±126 |
| 167244 | FS-93 | *Sambucus* sp.  Charcoal | 200±20 | Historic Navajo | AD 1731±66 |
| 267250 | FS-173 | Hardwood  Charcoal | 970±25 | Pueblo II-III | AD 1071±45 |
| 167243 | FS-90 | Hardwood Charcoal | 985±20 | Pueblo II | AD 1059±41 |
| 167249 | FS-109 | *Populus* sp.  Charcoal | 1245±20 | Pueblo I | AD 742±34 |
| 167247 | FS 98 | *Juniperus* sp.  Charcoal | 1690±20 | Basket-maker II | AD 356±28 |
| *18C/03107 | FS-116 | *Juniperus* sp.  Charcoal | 2010±30 | Basket-maker II | 25±75 BC |

**S4 Table. Pollen results.** Operations C-2 and C-4

| **Unit** | **C02** | **C02** | **C02** | **C02** | **C02** | **C02** | **C02** | **C04** | **C04** |
| --- | --- | --- | --- | --- | --- | --- | --- | --- | --- |
| **Depth (cm)** | 136 | 132 | 120 | 111 | 106 | 86 | 50 | 98 | 68 |
| **Direct Date** |  | 500 +/- 200 BC |  |  |  | 400 +/- 100 AD |  | 600 +/- 100 AD |  |
| **Specimen Number** | 2 | 3 | 4 | 5 | 6 | 8 | 9 | 13 | 14 |
| **Taxon** |  |  |  |  |  |  |  |  |  |
| *Ambrosia* | 5 | 7 | 3 | 6.5 | 10.5 | 7 | 12.5 | 10.5 | 16 |
| *Artemisia* | 1.5 | 4 | 4.5 | 3.5 | 6.5 | 4 | 8 | 9.5 | 6.5 |
| Asteraceae High Spine | 0.5 | 1.5 |  |  | 1 | 2.5 | 1.5 | 1 |  |
| *Solidago* | 3 | 6.5 | 5 | 8.5 | 3 | 2.5 | 9.5 | 5 | 9.5 |
| Brassicaceae |  |  |  |  |  | 1.5 | 1 | 0.5 | 0.5 |
| Cheno-Am | 42.5 | 42 | 55 | 52.5 | 39 | 37.5 | 35.5 | 38 | 49 |
| *Dalea* | 0.5 |  |  |  |  |  |  |  |  |
| *Ephedra nevadensis* | 0.5 |  | 1.5 |  |  |  |  | 3.5 | 0.5 |
| *Eriogonum* |  |  |  |  |  |  |  |  |  |
| Fabaceae | 1 |  |  | 0.5 | 0.5 | 0.5 |  | 0.5 |  |
| Lamiaceae |  |  |  |  | 1 |  |  |  |  |
| Liliaceae |  |  |  |  |  |  | 0.5 |  |  |
| *Plantago* |  |  |  |  | 0.5 |  |  |  |  |
| *Platyopuntia* | 0.5 |  |  |  |  |  |  |  |  |
| Poaceae | 6.5 | 5 | 8.5 | 4.5 | 7.5 | 3.5 | 3 | 4.5 | 3 |
| Polygonaceae |  |  | 0.5 | 1 | 0.5 | 1 | 0.5 | 0.5 | 0.5 |
| Rosaceae | 0.5 |  |  |  | 0.5 |  |  |  |  |
| *Sarcobatus* | 0.5 | 2 |  | 1.5 | 2.5 | 4 | 1 | 4.5 | 2 |
| Solanaceae |  | 0.5 |  | 0.5 |  |  |  |  |  |
| *Sphaeralcea* |  |  |  |  |  |  | 0.5 |  |  |
| *Typha* |  |  | 1 |  |  |  |  |  |  |
| *Celtis* | 0.5 |  |  |  |  |  |  |  |  |
| *Juniperus* | 20.5 | 8 | 4 | 3.5 | 3.5 | 8 | 6.5 | 4 | 4.5 |
| *Pinus* | 12 | 14 | 10.5 | 10 | 14.5 | 20 | 15 | 14 | 3 |
| *Quercus* | 0.5 | 2 |  | 1.5 |  |  | 1 |  |  |
| Rhamnaceae |  | 0.5 | 0.5 |  | 0.5 |  |  |  |  |
| *Salix* |  | 1.5 | 1.5 | 2 | 1 | 0.5 | 1 | 1.5 | 0.5 |
| Indeterminate | 4 | 5.5 | 4.5 | 4 | 7.5 | 7.5 | 3 | 2.5 | 4.5 |
| Total Pollen % | 100 | 100 | 100 | 100 | 100 | 100 | 100 | 100 | 100 |
| *Lycopodium* | 226 | 353 | 220 | 217 | 430 | 540 | 156 | 82 | 295 |
| **Concentration Value** | **1845** | **1510** | **2500** | **3898** | **1227** | **772** | **2673** | **5085** | **1413** |
| **Weight per Sample** | **10** | **7.82** | **7.58** | **4.93** | **7.9** | **10** | **10** | **10** | **10** |

**S5 Table.** **Pollen results.** Operations C-1 and C-3

| **Excavation Unit** | **C01** | **C01** | **C01** | **C01** | **C01** | **C03** | **C03** |
| --- | --- | --- | --- | --- | --- | --- | --- |
| **Stratum** | K | J | G (1&2) | D | C-4 | C-2 | C-1 |
| **Depth (cm)** | 115 | 105 | 85 | 70 | 28 | 55 | 30 |
| **Direct Date** | 916 +/- 100 AD |  |  | 916 +/- 100 AD | 1000 +/- 100 AD | 1799 +/-126 AD | 1800 +/-125 AD |
| **Specimen Number** | 12 | 17 | 18 | 11 | 10 | 16 | 15 |
| **Taxon** |  |  |  |  |  |  |  |
| *Ambrosia* | 13 | 4 | 4 | 14 | 12 | 8.5 | 6 |
| *Artemisia* | 6 | 8.5 | 4 | 6 | 5 | 11 | 1 |
| Asteraceae high spine | 1 |  | 3.5 |  | 0.5 |  | 3 |
| *Solidago* | 4 | 11.5 | 4 | 10 | 6 | 13.5 | 47.5 |
| Brassicaceae |  |  |  |  |  |  |  |
| Cheno-Am | 61.5 | 24 | 34.5 | 44 | 34.5 | 39 | 14 |
| *Dalea* |  |  | 0.5 |  |  |  |  |
| *Ephedra nevadensis* | 0.5 |  | 0.5 | 0.5 |  |  |  |
| *Eriogonum* |  |  |  |  |  |  | 5.5 |
| Fabaceae |  |  | 2.5 | 1 |  |  | 4.5 |
| Lamiaceae |  |  |  |  |  |  |  |
| Liliaceae |  |  |  |  |  |  |  |
| *Plantago* |  |  |  |  |  |  |  |
| *Platyopuntia* |  |  |  |  |  |  |  |
| Poaceae | 2.5 | 3.5 | 7.5 | 1 | 6 | 3.5 | 1.5 |
| Polygonaceae |  | 2.5 | 1.5 | 1 | 1 | 0.5 | 0.5 |
| Rosaceae |  | 0.5 |  |  |  |  |  |
| *Sarcobatus* | 1 |  | 1 | 0.5 | 1 | 1 |  |
| Solanaceae |  |  |  |  |  |  |  |
| *Sphaeralcea* |  |  |  |  |  |  |  |
| *Typha* |  | 0.5 |  |  |  |  |  |
| *Celtis* |  |  |  |  |  |  |  |
| *Juniperus* | 0.5 | 5 | 5.5 | 3.5 | 2.5 | 5 | 2 |
| *Pinus* | 6 | 35.5 | 25 | 10.5 | 29.5 | 11.5 | 3 |
| *Quercus* | 0.5 | 0.5 | 0.5 |  |  | 1 |  |
| Rhamnaceae |  |  |  |  |  |  |  |
| *Salix* | 0.5 | 0.5 | 0.5 | 2 |  |  | 4 |
| Indeterminate | 3 | 3.5 | 5 | 6 | 2 | 5.5 | 7.5 |
| Total Pollen % | 100 | 100 | 100 | 100 | 100 | 100 | 100 |
| *Lycopodium* | 112 | 123 | 176 | 60 | 200 | 228 | 72 |
| Concentration Value | 3723 | 3599 | 2455 | 6949 | 2085 | 2188 | 5903 |
| Weight per Sample | 10 | 9.42 | 9.65 | 10 | 10 | 8.36 | 9.81 |

**S6 Table. Macrobotanical samples collected as part of the Chaco Canyon Research Project.** Accession numbers (form number plus item number) are in the two columns on the left**.** Samples stored in the Paleoethnobotanical Laboratory, University of Cincinnati, Cincinnati, OH, USA

Accession numbers (form number plus item number) are in the two columns on the left**.** Currently all specimens are housed in the Paleoethnobotanical Laboratory, 731 Rieveschl Hall at the University of Cincinnati, Cincinnati, OH 45221. When renovations are completed, the wood samples listed will be moved to the Xylarium of the Margaret H. Fulford Herbarium (CINC), 620 Rieveschl Hall, University of Cincinnati, Cincinnati, OH 45221. The seeds from this study will be housed in the Carpological section of CINC.

| **Form #** | **Item #** | **Op.** | **Field Sample** | **Depth** | **Taxonomic Name** | **Common Name** | **Plant Part** | **Weight (g)** |
| --- | --- | --- | --- | --- | --- | --- | --- | --- |
| 10001 | 001 | CO1 | FS88 | 34-50cmbd | *Sambucus* cf. *racemosa* | Elm | charcoal | 2.27 |
| 10002 | 001 | CO1 | FS87 | 39-56cmbd | *Pinus* sp. | Pine | charcoal | 0.04 |
| 10003 | 001 | CO1 | FS90 | 53-57cmbd | Hardwood |  | charcoal | 0.04 |
| 10004 | 001 | CO1 | FS93 | 55-71cmbd | *Sambucus* cf. *racemosa* | Elderberry | charcoal | 3.45 |
| 10005 | 001 | CO1 | FS89 | 57-72cmbd | *Sambucus* cf. *racemosa* | Elderberry | charcoal | 0.43 |
| 10006 | 001 | CO1 | FS95 | 71-112cmbd | *Sambucus* cf. *racemosa* | Elderberry | charcoal | 0.35 |
| 10006 | 002 | CO1 | FS95 | 71-112cmbd | Spermatophyte |  | tissue | 0.82 |
| 10007 | 001 | CO1 | FS92 | 90cmbd | Hardwood |  | charcoal | 0.03 |
| 10008 | 001 | CO2 | FS107 | 150cmbd | Coal? |  | charcoal? | 0.21 |
| 10008 | 002 | CO2 | FS107 | 150cmbd | Spermatophyte |  | tissue | 0.11 |
| 10009 | 001 | CO3 | FS96 | 42-55cmbd | Hardwood |  | charcoal | 0.06 |
| 10010 | 001 | CO2 | FS110 | 125cmbd | Conifer |  | charcoal | 0.4 |
| 10011 | 001 | CO3 | FS97 | 60cmbd | *Sambucus* cf. *racemosa* | Elderberry | charcoal | 0.15 |
| 10012 | 001 | CO3 | FS98 | 60-92cmbd | *Juniperus* sp. | Juniper | charcoal | 0.18 |
| 10013 | 001 | CO4 | FS117 | 28-37cmbd | *Juniperus* sp. | Juniper | charcoal | 0.26 |
| 10014 | 001 | CO4 | FS114 | 84cmbd | *Salix* sp. | Willow | charcoal | 0.35 |
| 10015 | 001 | CO4 | FS116 | 98cmbd | *Juniperus* sp. |  | charcoal | 0.04 |
| 10016 | 001 | CO4 | FS175 | 106cmbd | *Pinus* sp. | Pine | charcoal | 0.15 |
| 10017 | 001 | CO4 | FS173 | 138cmbd | Hardwood |  | charcoal | 0.14 |
| 10018 | 001 | CO4 | FS174 | 153cmbd | Conifer |  | charcoal | 0.08 |
| 10019 | 001 | EO2 | FS176 | 80cmbd | Spermatophyte |  | tissue | 0.05 |
| 10020 | 001 | A06 | 100 | 108 cmbd | *Portulaca oleracea* | Purslane | seeds | <.01 |
| 10020 | 002 | A06 | 100 | 108 cmbd | Amaranthaceae | Amaranth | seed | <.01 |
| 10020 | 003 | A06 | 100 | 108 cmbd | *Atriplex* sp. | Saltbush | seed | <.01 |
| 10020 | 004 | A06 | 100 | 108 cmbd | *Dysphania* sp. | Chenopodium | seed | <.01 |
| 10020 | 005 | A06 | 100 | 108 cmbd | coprolites |  |  | 0.33 |
| 10021 | 001 | A09 | 102 | 185 cmbd | Hardwood |  | charcoal | 0.12 |
| 10022 | 001 | A09 | 103 | 240 cmbd | Hardwood? |  | charcoal | 0.14 |
| 10023 | 001 | A09 | 104 | 165 cmbd | coal? |  |  | 0.93 |

**S6 Table. Continued.**

| **Form #** | **Item #** | **Op.** | **Field Sample** | **Depth** | **Taxonomic Name** | **Common Name** | **Plant Part** | **Weight (g)** |
| --- | --- | --- | --- | --- | --- | --- | --- | --- |
| 10024 | 001 | A09 | 105 | 115 cmbd | Hardwood |  | charcoal | 0.17 |
| 10025 | 001 | A09 | 106 | 80 cmbd | Spermatophyte |  | tissue | 0.26 |
| 10026 | 001 | C01 | 91 | 85 cmbd | *Pinus* sp. | Pine | charcoal | 0.38 |
| 10027 | 001 | C01 | 94 | 92-120 cmbd | Spermatophyte |  | tissue | 0.01 |
| 10028 | 001 | C01 | 133 | 27-32 cmbd | Ulmus sp. ?? | Elm | charcoal | 0.04 |
| 10029 | 001 | C02 | 108 | 115 cmbd | *Morus* sp. | Mulberry | charcoal | 1.37 |
| 10030 | 001 | C02 | 109 | 142 cmbd | *Populus* sp. | Cottonwood | charcoal | 1.49 |
| 10031 | 001 | C02 | 111 | 100 cmbd | Spermatophyte |  | tissue | 0.42 |
| 10032 | 001 | C02 | 112 | 125 cmbd | Unknown |  | charcoal | 3.09 |
| 10033 | 001 | C02 | 113 | 100 cmbd | *Pinus* sp. | Pine | charcoal | 1.39 |
| 10034 | 001 | C03 | 99 | 91 cmbd | Hardwood? |  | charcoal | 0.05 |
| 10035 | 001 | C04 | 115 | 107 cmbd | Conifer |  | charcoal | 0.01 |
| 10036 | 001 | E01 | 101 | 178 cmbd | Hardwood |  | charcoal | 0.05 |
| 10037 | 001 | C02 | Float (light) | 115 cmbd | Hardwood |  | charcoal | 0.06 |
| 10037 | 002 | C02 | Float (light) | 115 cmbd | *Verbena* sp. | Verbena | seed | <.01 |
| 10037 | 003 | C02 | Float (light) | 115 cmbd | Spermatophyte |  | tissue | 2.62 |
| 10037 | 004 | C02 | Float (light) | 115 cmbd | Fungal |  | spore | <.01 |
| 10037 | 005 | C02 | Float (light) | 115 cmbd | cf. *Atriplex* sp. | Saltbush | seed | <.01 |
| 10037 | 006 | C02 | Float (light) | 115 cmbd | unknown |  | exocarps | 0.01 |
| 10038 | 001 | C02 | Float (heavy) | 115 cmbd | Spermatophyte |  | tissue | 0.11 |
